# Habitual caffeine consumption moderates the antidepressant effect of dorsomedial intermittent theta-burst transcranial magnetic stimulation

**DOI:** 10.1177/02698811211058975

**Published:** 2021-12-06

**Authors:** Andreas Frick, Jonas Persson, Robert Bodén

**Affiliations:** 1 The Beijer Laboratory, Department of Neuroscience, Psychiatry, Uppsala University, Uppsala, Sweden; 2 Department of Neuroscience, Psychiatry, Uppsala University, Uppsala, Sweden

**Keywords:** Adenosine, depression, caffeine, rTMS, coffee, repetitive transcranial magnetic stimulation

## Abstract

**Background:**

Potentiating current antidepressant treatment is much needed. Based on animal studies, caffeine may augment the effects of currently available antidepressants.

**Objective:**

Here, we tested whether habitual caffeine consumption moderates the antidepressant effects of repetitive transcranial magnetic stimulation (rTMS) using intermittent theta-burst stimulation (iTBS).

**Methods:**

Forty patients with current depressive episodes were randomized to active iTBS (*n* = 19) or sham treatment (*n* = 21; shielded side of the coil and weak transcutaneous electrical stimulation) delivered two times per day for 10–15 weekdays. Neuronavigated stimulation was applied to the dorsomedial prefrontal cortex. Symptom improvement was measured using change in self-reported Montgomery-Åsberg Depression Rating Scale (MADRS) scores. Pretreatment habitual caffeine consumption was quantified using self-reports of number of cups of coffee and energy drinks consumed the 2 days before the treatment starts.

**Results:**

Habitual caffeine consumption was associated with symptom improvement following active iTBS (*r* = 0.51, 95% confidence interval (CI): 0.08–0.78, *p* = 0.025) but not following sham treatment (*r* = −0.02, 95% CI: −0.45 to 0.42, *p* = 0.938). A multiple regression analysis corroborated the findings by showing a significant caffeine consumption × treatment group interaction (β = 0.62, *p* = 0.043), but no main effects of treatment group (β = 0.22, *p* = 0.140) or caffeine consumption (β = −0.01, *p* = 0.948). No group differences in pretreatment symptom scores or caffeine consumption were detected (*p* values > 0.86).

**Conclusion:**

Habitual caffeine consumption moderated the antidepressant effect of dorsomedial iTBS, consistent with caffeine improving antidepressant pharmacological treatments in animals. Caffeine is an antagonist of adenosine receptors and may enhance antidepressant effects through downstream dopaminergic targets.

Mood disorders are highly prevalent, impairing, and costly psychiatric conditions associated with premature death and individual suffering (Kessler et al., 2012; Whiteford et al., 2013). Patients with mood disorders often suffer from long-lasting depressive episodes, characterized by lowered mood or loss of interest or pleasure during a period of at least 2 weeks and often associated with fatigue (American Psychiatric Association, 2013). Current first-line treatment options for depressive episodes include cognitive-behavioral therapy (CBT) and selective serotonin re-uptake inhibitors (SSRIs), but response rates are only around 50% (DeRubeis et al., 2005; Rush et al., 2006), which has prompted research into other treatment options and ways to potentiate current treatments (Månsson et al., 2021).

Repetitive transcranial magnetic stimulation (rTMS) with high- or low-frequency stimulation, or the more feasible short-duration intermittent theta-burst stimulation (iTBS), has been found to be effective in depressive patients not responding to CBT or drugs for depression (Blumberger et al., 2018; Sehatzadeh et al., 2019). The most established target for antidepressant rTMS is the dorsolateral prefrontal cortex (dlPFC) (Downar and Daskalakis, 2013; Sehatzadeh et al., 2019), but with remission rates of 16% and response rates of 25% in patients with treatment-resistant depression, there is still an unmet need for further treatment options, calling for new treatment targets. The dorsomedial prefrontal cortex (dmPFC) has been suggested as an alternative target because of its involvement in emotion regulation and reward processing (Downar and Daskalakis, 2013), which are compromised in depressive patients (Halahakoon et al., 2020). Reward processing involves dopaminergic signaling (Wise and Rompre, 1989) and, in accordance, depression is associated with reduced reward-related dopamine neurotransmission in the ventral striatum (Nestler and Carlezon, 2006). Interestingly, studies in rodents have shown that neuromodulation of the mPFC increases striatal dopamine signaling (Hill et al., 2018), which may suggest that dmPFC rTMS could increase dopaminergic signaling and thereby have an antidepressant effect. However, clinical trials have not provided evidence for stronger antidepressant effects of active dmPFC rTMS than sham treatment on a group level (Bodén et al., 2021; Dunlop et al., 2020), which opens for questions of treatment responses being contingent upon individual variation in hitherto unknown factors. We here suggest that one of these factors may be caffeine consumption.

Supporting this notion, animal studies have shown that caffeine potentiates the effects of currently available antidepressant drugs (Szopa et al., 2016), but despite these findings, the augmenting effects of caffeine on antidepressant treatment have not been examined in humans. Caffeine is an adenosine receptor antagonist, and its psychostimulant effects, which may counteract the anergia of depression (Lopez-Cruz et al., 2018), involve caffeine antagonism of A2A receptors and downstream increased dopamine D2 signaling (Lazarus et al., 2011). There are also epidemiological findings of habitual caffeine consumption reducing risk of depression (Lucas, 2011; Smith, 2009). Hence, given the purported dopaminergic effects of dmPFC stimulation, there are reasons to believe that caffeine could interact with dmPFC rTMS to augment dopaminergic signaling and increase the antidepressant effects.

We recently performed a clinical trial of iTBS of the dmPFC in depression and schizophrenia (Bodén et al., 2021). The results of this study indicated no significant advantage of dmPFC iTBS over sham treatment on overall depressive symptoms in the group of patients with depression. Here, in a follow-up analysis to our recent study, we tested the hypothesis that individual differences in habitual caffeine consumption moderates the antidepressant treatment effects of dmPFC iTBS.

## Methods

The current sample is a subset of the patients in a previously published randomized, double-blind, sham-controlled trial examining the effects of dmPFC iTBS on anhedonia symptoms in patients with either depressive disorder or schizophrenia (Bodén et al., 2021). This study constitutes a follow-up analysis of the moderating effects of caffeine on antidepressant treatment outcome, including only the patients with depression. The study was approved by the regional ethical review board and the Swedish Medical Products Agency. All participants provided written informed consent before entering the study.

For a full description of the study protocol, we refer to Bodén et al. (2021). In brief, 40 patients with an ongoing depressive episode were recruited from the psychiatric outpatient clinic at Uppsala University Hospital and randomized to active iTBS (*n* = 19) or sham treatment (*n* = 21) delivered twice-daily for 10–15 weekdays. Inclusion criteria consisted of 18–59 years of age, having a uni- or bipolar depression diagnosis verified by the Mini International Neuropsychiatric Interview (MINI) (Sheehan et al., 1998), ⩽40 points on the Motivation and Pleasure Scale—Self-Report (Llerena et al., 2013), and stable pharmacotherapy the past month. Exclusion criteria comprised epilepsy, magnetic sensitive metals implanted in the head or within 30 cm of the treatment coil, implanted devices activated/controlled by physiological signals, conditions that seriously increases the risk of non-compliance or loss of follow-up, active substance use disorder, and pregnancy. Alcohol and drug use was assessed using the Alcohol Use Disorders Identification Test (AUDIT) (Bergman et al., 1994), the Drug Use Disorders Identification Test (DUDIT) (Berman et al., 2005), and urine screening test for illicit drugs. Likewise, pregnancy was assessed by urine screening test.

### Treatment

TMS coil placement to target the dmPFC was determined through a magnetic resonance imaging–based neuronavigation system (TMS Navigator, Localite, Bonn, Germany) and a T1-weighted anatomical brain scan for 30 of the patients. For the remaining 10 subjects, coil placement was defined as 25% of the nasion-inion distance in the middle-line of the scalp. Treatment was delivered using a magnetic stimulator Magpro ×100 with Magoption with a Cool-DB80 A/P, and a combined active/sham coil with identically looking sides. The sham side of the coil is shielded, preventing approximately 95% of the magnetic field to reach the participant. Each participant’s randomization code was entered into the stimulator research software by the operator, and a position sensor in the coil signaled which side (active/sham) was to be angled toward the patient.

Active treatment consisted of iTBS with 20 trains of stimulation with right-left current direction and 20 trains of stimulation with left-right direction. Each train had 2 s stimulation and 8 s off, with the stimulation being 10 bursts at 5 Hz and each burst having 3 biphasic pulses at 50 Hz. A total of 1200 pulses/session were administered. Stimulation was applied at an intensity of 90% of the resting motor threshold, determined as the lowest intensity eliciting a visual muscle contraction in the foot when applied to the medial motor cortex for the extensor halluces longus. Intensity was ramped up during initial treatments. Patients came for twice-daily treatment sessions during weekdays with an interval of 15 min between sessions, aiming for a total of 10 days of treatments, where at least 50% of the trains reached the predetermined treatment intensity (90% of motor threshold). Treatment was prolonged with 1 day, to a maximum of 15 days, for each treatment day that did not meet these criteria. The sham treatment consisted of an identical stimulation protocol but with the shielded side of the coil angled toward the participant. Each participant had two transcutaneous electrical nerve stimulation (TENS) electrodes placed medially on the forehead under the coil, and in the sham group, a maximum current of 4 mA, scaled by stimulation intensity, was delivered synchronous with the magnet pulse to mimic the sensation of magnetic stimulation. To evaluate blinding of patients, they were asked to guess the treatment allocation following the first, fifth, and last treatment day (Bodén et al., 2021). We also assessed blinding of the nurses operating the magnetic stimulator.

### Clinical outcome measures

Depressive symptoms were measured using the self-reported Montgomery-Åsberg Depression Rating Scale (MADRS-S) (Svanborg and Åsberg, 1994) administered at baseline and following the last treatment session.

### Caffeine consumption

Pretreatment habitual caffeine consumption was quantified as the mean of self-reported number of cups of coffee and energy drinks consumed the day before and 2 days before the treatment starts. Caffeine content in a cup of coffee and an energy drink is similar, about 80–100 mg. We here multiplied the number of drinks with 90 to calculate the amount of caffeine consumed.

### Statistical analyses

Pearson product–moment correlations were used to test for associations between pretreatment caffeine consumption and symptom improvement in the whole sample and separately within treatment groups. To test for differential effects of caffeine consumption in the two treatment groups, we performed multiple regression analyses of symptom improvement (MADRS-S pre–post differential score) with Caffeine, Group, and their interaction (Caffeine × Group) as predictors. The Caffeine × Group interaction tested for the moderating effect of pretreatment caffeine consumption on treatment group’s effect on outcome, that is, a significant interaction effect would indicate a differential augmenting or attenuating effect of caffeine consumption on treatment outcome.

## Results

See Table 1 for clinical and demographic characteristics. No differences were detected between treatment groups on pretreatment depressive symptoms or habitual caffeine consumption (*p* values *>* 0.15). No associations between pretreatment habitual caffeine consumption and symptom severity or resting motor threshold were detected within or across the groups (*p* values > 0.23). As previously reported, patients were more likely than chance to guess treatment allocation after the first day, but not after the fifth and the last treatment days (Bodén et al., 2021). The nurses operating the magnetic stimulator were not successfully blinded to treatment allocation.

**Table 1.** Participant characteristics in the intermittent theta-burst transcranial magnetic stimulation (iTBS) and sham treatment groups, including statistical tests of group differences.

	iTBS (*n* = 19)	Sham (*n* = 21)	Statistic	*p*
Demographic and clinical information				
Age, years; mean (SD)	30.1 (10.3)	28.7 (8.8)	*t* = 0.460	0.648
Sex, women; *n* (%)	10 (52.6%)	11 (52.4%)	χ^2^ < 0.001	0.987
Motor threshold; mean (SD)	54.5 (10.3)	50.3 (9.5)	*t* = 1.338	0.189
Daily caffeine consumption (cups/drinks); mean (SD)	1.3 (1.6)	1.4 (1.6)	*t* = 0.169	0.866
Depressive symptoms baseline—MADRS-S; mean (SD)	30.2 (8.6)	30.2 (8.0)	*t* = 0.031	0.976
Depressive symptom improvement—MADRS-S; mean (SD)	4.5 (7.6)	1.9 (4.7)	*t* = 1.346	0.186
Diagnosis, *n* (%)			χ^2^ = 0.902	0.342
Bipolar disorder	1 (5.3%)	3 (14.3%)		
Major depressive disorder	18 (94.7 %)	18 (85.7%)		
Ongoing antidepressant treatment, *n* (%)	17 (89.5%)	15 (71.4%)	χ^2^ = 2.030	0.154
Serotonin reuptake inhibitors	6	3		
Serotonin-norepinephrine reuptake inhibitors	8	8		
Other antidepressant^a^	9	8		
Ongoing dopamine D2 receptor antagonist treatment, n (%)	4 (21.1%)	5 (23.8%)	χ^2^ = 0.043	0.835

MADRS-S: Montgomery Asberg Depression Rating Scale—Self-Report.

a iTBS-group: mirtazapine (*n* = 3), bupropione (*n* = 3), vortioxetine (*n* = 2), agomelatine (*n* = 1); sham group: mirtazapine (*n* = 6), selegiline (*n* = 1), vortioxetine (*n* = 1).

**Figure 1.**
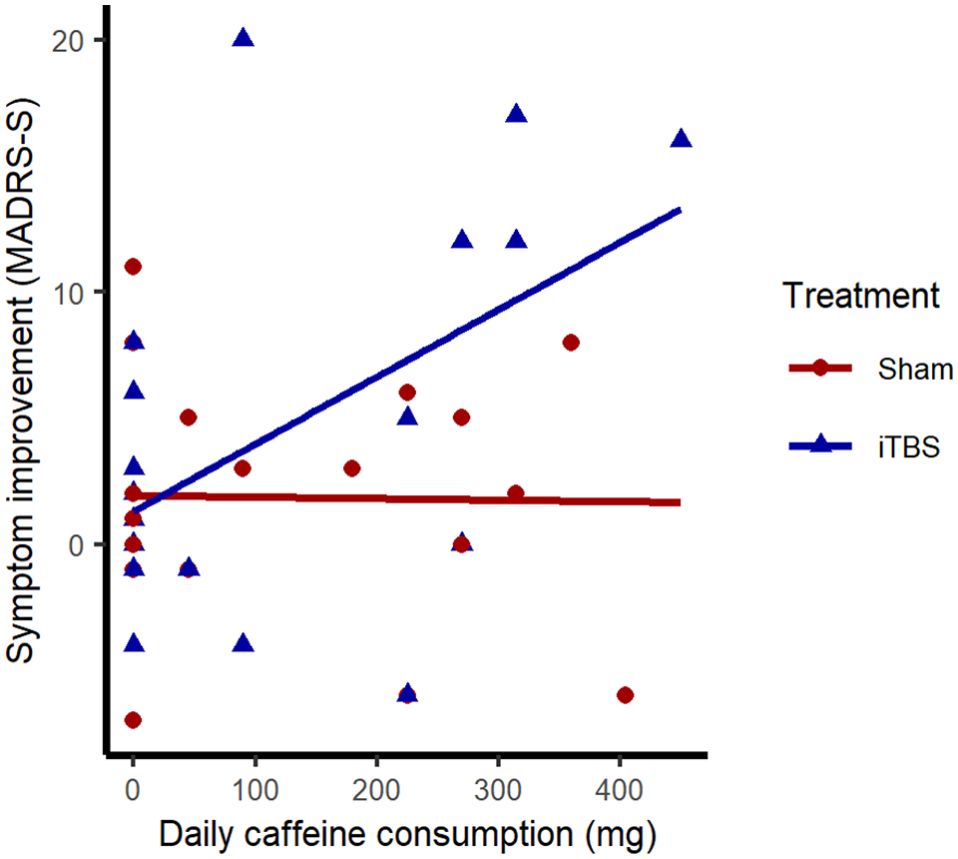
Scatterplot and linear fit lines for associations between pretreatment habitual caffeine consumption and depressive symptom improvement within active intermittent theta-burst stimulation (iTBS) and sham treatment groups. Depressive symptom improvement indexed by change in Montgomery Åsberg Depression Rating Scale—Self-Report (MADRS-S) scores during treatment.

Across the two treatment groups, pretreatment habitual caffeine consumption was weakly related to symptom improvement (*r* = .28, 95% CI: −0.03 to 0.54, *p* = 0.080). Within-group correlations revealed that only patients receiving active iTBS (*r* = .51, 95% CI: 0.08–0.78, *p* = 0.025) and not sham treatment (*r* = −0.02, 95% CI: −0.45 to 0.42, *p* = 0.938) showed greater depressive symptom improvement with larger pretreatment caffeine consumption (Figure 1). A multiple regression analysis (*F*(3, 36) = 3.426, *p* = 0.027, adjusted *R*^2^ = 0.16) identified an interaction effect between pretreatment habitual caffeine consumption and treatment group on depressive symptom improvement (β = 0.62, *p* = 0.043), but no main effects of group (β = 0.22, *p* = 0.140) or caffeine consumption (β = −0.01, *p* = 0.948). Age, sex, and smoking have been linked to caffeine consumption (Nehlig, 2018). We therefore conducted a sensitivity analysis including these variables in the multiple regression, which indicated that the interaction between caffeine consumption and treatment remained (β = 0.66, *p* = 0.042) and that age (β = 0.01, *p* = 0.934), sex (β = 0.15, *p* = 0.337), and smoking (β = 0.01, *p* = 0.948) did not contribute to improvement.

## Discussion

In this follow-up analysis of our recent double-blind, sham-controlled trial of dmPFC iTBS (Bodén et al., 2021), pretreatment habitual caffeine consumption predicted the antidepressant effect of active compared to sham dorsomedial iTBS in patients with depression. This effect was only present in the active treatment group and not in patients receiving sham treatment, pointing to the interaction between iTBS and caffeine consumption as being important for an antidepressant effect.

The potentiating effect of caffeine on iTBS is consistent with caffeine improving antidepressant pharmacological treatments in animals (Szopa et al., 2016) and mirror findings of concurrent psychostimulant use potentiating antidepressant effects of dlPFC rTMS (Hunter et al., 2019), but we are not aware of any previous human trial examining caffeine as an adjuvant to antidepressant treatment. The exception being electroconvulsive therapy, where caffeine may improve the effect by increasing seizure time (Bozymski et al., 2018). Other psychostimulants (e.g. amphetamine derivatives and modafinil) augment antidepressant effects (Hunter et al., 2019; Pary et al., 2015), but the potential for misuse and side effects of these compounds need to be managed. Similarly, increasing dopaminergic signaling using dopamine agonists (e.g. pramipexole) have also been found to be effective adjuvants in treatment-resistant depression (Cusin et al., 2013), but the direct dopaminergic action gives rise to similar side effects and potential misuse as psychostimulants, such as amphetamine. Overcoming these limitations, but retaining the positive augmenting effects, is thus desirable, and in this respect, perhaps caffeine can be a more favorable alternative.

We can only speculate about the mechanisms underlying the potentiating antidepressant effect of caffeine on iTBS, but argue that caffeine’s antagonism of adenosine receptors and downstream dopaminergic influences are likely candidates. Adenosine acts as a modulator of neural activity, mainly through the adenosine A1 and A2A receptors (Fredholm et al., 2005), and caffeine is a nonselective antagonist of these receptors. Converging evidence implicates the involvement of adenosine receptors in mood disorders (Ortiz et al., 2015; Yamada et al., 2014). For example, animals overexpressing A2A receptors show more depressive-like behavior (Coelho et al., 2014) and A2A receptors are upregulated following chronic mild stress (Crema et al., 2013), used as an experimental model of depression. Conversely, genetic knock-out or pharmacological blockade of A2A receptors alleviates depression-like symptoms (Kaster et al., 2015; Ortiz et al., 2015; Yacoubi et al., 2009; Yamada et al., 2014). Thus, nonselective antagonism of adenosine receptors may have a weak antidepressant effect that is evident when combined with iTBS or other antidepressant treatments as seen in animals (Szopa et al., 2016). One of the proposed antidepressant mechanisms of A2A receptor antagonism is its positive effect on dopaminergic signaling (Ferré et al., 2008; Lopez-Cruz et al., 2018), thereby reversing the reduced reward-related dopamine neurotransmission associated with depression (Nestler and Carlezon, 2006). Interestingly, administration of L-DOPA, the precursor of dopamine, increased synaptic plasticity when combined with a TMS paradigm, most probably through dopaminergic action at dopamine D1 receptors, because administration of the D2-class agonist pramipexole did not affect synaptic plasticity (Enomoto et al., 2015). In addition, caffeine-induced arousal, mediated by antagonism of A2A and downstream-increased dopamine D2 signaling (Lazarus et al., 2011), counteracts the anergia of depression and may also be involved in the antidepressant response (Lopez-Cruz et al., 2018). Thus, the potentiating antidepressant effect of caffeine may be mediated by downstream effects on dopaminergic signaling at D1 receptors leading to increased synaptic plasticity or at D2 receptors targeting anergia, or a combination of the two.

There are some limitations of this study that deserve mentioning. First, the observational nature of this study with regard to caffeine consumption means that we cannot rule out confounding common factors associated with both higher caffeine consumption and predisposing for beneficial iTBS effect. These potential confounders may include genetic factors, as caffeine consumption has a strong genetic component (Nehlig, 2018). Future experimental studies should replicate and further investigate potential mechanisms, for example, regarding dopaminergic signaling. Second, the relatively small sample size warrants some caution until future replication has been performed. Third, caffeine consumption was self-reported, which may open for recall bias, although the short time-frame of recall (yesterday and the day before yesterday) makes large deviations from actual consumption less likely and a potential recall bias would nevertheless probably not co-vary with treatment allocation. Moreover, the timing of caffeine consumption in relation to the rTMS sessions lacks temporal precision, and although our measure is one of habitual consumption, we cannot rule out acute effects of caffeine. Also, caffeine consumption was calculated from coffee and energy drink intake, which left out potential sources of caffeine (e.g. tea), although studies have shown that the absolute majority of caffeine consumed comes from coffee (Frary et al., 2005). The inclusion of blood caffeine concentration would have added information to the analyses but was unfortunately not available. Regarding the treatment protocol, the 10–15 days of stimulation may be too short to induce full treatment effect, and our twice-daily iTBS with a 15-min intersession interval may not double the improvement rate. This concern is highlighted by the dramatic effects of the recent pilot study with accelerated iTBS using a 50 min intersession interval (Cole et al., 2020), compared to the more modest effects reported in another recent open-label study using a 15 min intersession interval (Baeken et al., 2017). Furthermore, the nurses operating the magnetic stimulator were not blinded to treatment allocation. Nevertheless, patients were not more likely than chance to guess correct treatment after the fifth and last days of treatment.

In conclusion, habitual caffeine consumption augmented the antidepressant effect of dorsomedial iTBS, which opens for future trials systematically testing the potentiating effect of caffeine and more selective adenosine receptor ligands on antidepressant treatments in the clinic.
